# A Prospective Observational Cohort Study to Analyze the Etiology of Revision Lumbar Spine Surgery at a Tertiary Care Spine Institute

**DOI:** 10.7759/cureus.104001

**Published:** 2026-02-20

**Authors:** Mikeson Panthackel, Bharat R Dave, Ravi Ranjan Rai, Ajay Krishnan, Shivanand C Mayi, Mirant B Dave, Arjit Vashishtha, Amritesh Singh, Saurabh S Kulkarni, Yogenkumar Adodariya

**Affiliations:** 1 Spine Surgery, Stavya Spine Hospital and Research Institute, Ahmedabad, IND; 2 Spine, Bhavnagar Institute Of Medical Science, Bhavnagar, IND; 3 Spine, Stavya Spine Hospital and Research Institute, Ahmedabad, IND; 4 Orthopaedics, Geetanjali Medical College and Hospital, Eklingapura, IND; 5 Orthopaedics, University College Of Medical Sciences &amp; Guru Tej Bahadur Hospital, Delhi, IND; 6 Orthopedics, Mahatma Gandhi Mission Medical College and Hospital, Chhatrapati Sambhajinagar, IND; 7 Spine, Stavya Spine Hospital and Research Institute, Ahmedhabad, IND

**Keywords:** adjacent segment disease, asd, etiology, lumbar spine surgery, recurrent stenosis, revision outcomes, revision spine surgery

## Abstract

Introduction

Revision lumbar spine surgery is an increasing challenge, often necessitated by adjacent segment disease (ASD), recurrent stenosis, or implant-related complications. This study aimed to analyze the etiology and short-term outcomes of revision lumbar surgeries at a tertiary spine center.

Materials and methods

A prospective observational study was conducted at our institute, including all revision lumbar surgeries performed during the defined study period. Data collected included patient demographics, details of the index surgery, presenting complaints, indications for revision, characteristics of the revision procedure, and six-month postoperative outcomes (Visual Analogue Score (VAS) and Oswestry Disability Index (ODI)).

Results

A total of 103 revision surgeries (mean age 58.3 years; 54 females, 49 males) were included. Those in Group A were revised within two years (n=50, 49%); Group B were revised between two to five years (n=25, 24%); and Group C were revised more than five years after the index surgery (n=28, 27%). In Group A, revisions were predominantly in patients who had fusion procedures in the first surgery. In contrast, Group C had more patients whose index procedure was non-fusion. Analysis of the preoperative radiographs of previous surgeries revealed overlooked ASD, foraminal stenosis, and inadequately decompressed stenotic segments. Statistically significant improvement in pain and functional outcomes was noted at the six-month follow-up.

Conclusion

Prevention of early revision lumbar surgery requires adequate decompression during the primary procedure, identification and prophylactic treatment of partially stenotic adjacent levels, and recognition and decompression of the foraminal stenosis.

## Introduction

The number of spinal surgeries has seen an upward trend in recent times due to sedentary lifestyles, improved imaging modalities, and increased life expectancy [1,2]. Surgical treatment usually involves decompression, either alone or combined with fusion. Recent advances, like minimally invasive surgeries and 3D navigation, have also contributed to this increase by improving safety in spinal surgeries and patient confidence [3].

However, this has also contributed to a higher incidence of failed back surgery syndrome (FBSS) and revision lumbar spine surgeries. Failures of lumbar spine surgery could be divided into early and late failures. Early surgical failures (postoperative patients with no symptomatic relief or less than three weeks) might result from insufficient decompression, iatrogenic lumbar instability, or infections [4]. Late surgical failures (greater than three weeks) are a result of recurrent disc prolapse, acquired instability or adjacent level degeneration [5]. Multiple factors have been associated with revision spinal surgeries, including type of procedure, obesity, personal habits (smoking, alcoholism), and long fusion constructs [6].

Owing to the complexity of the procedure and the increased number of levels involved, revision lumbar surgeries have been associated with poorer outcomes as compared to the primary or index surgeries [7]. The goal of this study was to identify the various causes of revision lumbar spine surgery at our institute and find the short-term outcome. This study would also help us to identify the factors (e.g., smoking, BMI, diabetes (DM), other comorbidities) commonly associated with revision.

## Materials and methods

Study design and setting

Prospective, observational study conducted at a tertiary care center.

Study population

All patients undergoing revision lumbar spine surgery at our institute (Stavya Spine Hospital and Research Institute, Ahmedabad, Gujarat, India) between December 2023 and June 2024 were included. Inclusion criteria were surgeries at the previously operated same level or adjacent levels, and cases that had extension of fixation to the dorsal level. Cases with prior endoscopic, minimally invasive, or open spinal surgery were considered. Exclusion criteria were age <18 or >85 years, planned implant removal, and cervical or thoracic spinal surgeries.

Ethical considerations

The study was approved by the institutional Ethics Committee of the Stavya Spine Hospital and Research Institute (approval no. SSHRI/CS/NS/Revision S x 6 months/BRD/66/11.23), and registered with the Clinical Trial Registry of India (CTRI no. CTRI/2024/01/061208). As this is a prospective study, informed consent was taken from all patients.

Data collection and evaluation

Demographics, comorbidities, Body Mass Index (BMI), details of primary and subsequent surgeries, presenting complaints, preoperative (preop) and postoperative (postop) imaging of the first surgery (if available), and preop imaging of the present surgery were recorded. Implant position, residual disc (inadequate decompression), recurrent disc, residual symptoms, and pain-free interval after first surgery were assessed.

Definitions

1) Residual disc: Persistent symptoms without significant pain-free interval; 2) Recurrent disc: Symptom recurrence after ≥6 months of pain-free interval [8].

Surgical technique

Minimally invasive Wiltse approach [9] with tubular retractors was used for limited decompression or short-segment fusion. Standard midline open approach was used for implant removal or fixation extension.

Outcomes and follow-up

Short-term outcomes were assessed using the Visual Analog Scale (VAS) [10] and the Oswestry Disability Index (ODI) [11,12] scores. Patients were followed for six months, with outcomes recorded at final follow-up. Outcome scores were recorded in the outpatient clinic by an assessor blinded to the study objectives.

Statistical analysis

The chi-square test was applied to assess associations between categorical variables, while independent t-tests or Mann-Whitney U tests compared continuous outcomes between groups. Correlation between continuous parameters, such as BMI and time to revision, was analyzed using Spearman’s rank correlation. For the analysis, IBM SPSS Statistics for Windows, Version 20 (Released 2011; IBM Corp., Armonk, New York, United States). A p-value <0.05 was considered statistically significant.

## Results

Out of the 1,266 surgeries in the institution during the study period, 103 were revision lumbar spine surgeries. The mean age of patients undergoing revision surgery was 58.29. The gender distribution was 54 female patients and 49 male patients. The patient characteristics are presented in Table 1.

**Table 1:** Table showing the epidemiological data of the patients included in our study DM: Diabetes Mellitus; HTN: Hypertension; TSH: hypothyroidism; HTN+TSH: Hypertension and hypothyroidism. All the values are presented either in mean±SD or N (%) format.

Category		Summary
Age		Mean ± SD: 58.3 ± 12.7, Range: 20-80
Gender distribution	Female	54 (52.4%)
	Male	49 (47.6%)
BMI		Mean ± SD: 27.2 ± 4.1
Comorbidities	DM:	35 (35.7%)
	HTN:	33 (33.7%)
	TSH:	4 (4.0%)
	HTN+TSH	1 (1.0%)
	Hypoparathyroidism	1 (1.0%)
	No comorbidities:	29 (24.6%)
Smoking/Alcohol history	No	100 (97.1%)
	Smoking (minor cases)	3 (2.9%)

Radiculopathy (60%) represented the predominant clinical indication for revision surgery, followed by axial back pain (26.5%), neurogenic claudication (11.7%), and motor deficits (3.9%). The most common comorbidity encountered was diabetes mellitus.

**Figure 1:**
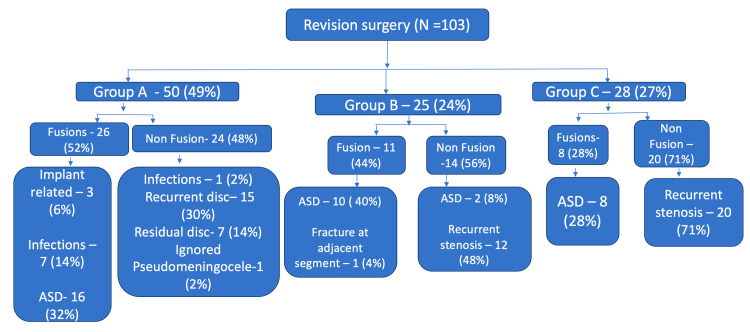
Distribution of the causes for revision lumbar surgeries across patient groups (n=250) All the values given in format N(%). For groups, the percentages are calculated based on the revision surgery. Percentages for divisions and subdivisions within each group were calculated using the total number of patients in that respective group as the denominator. Group A: revision surgery within two years of primary surgery; Group B: revision surgery between two and five years after primary surgery; Group C: revision surgery more than five years after primary surgery. ASD: Adjacent Segment Disease; Fusion: primary surgery involving spinal fusion; Non-fusion: primary surgery without fusion. Image credit: Created by Mikeson P using Microsoft PowerPoint (Microsoft, Redmond, Washington, US).

A total of 103 revision surgeries were analyzed, comprising 45 fusion and 58 non-fusion procedures. Patients were categorized into three groups based on the interval between the index and revision surgeries: Group A, revisions performed within two years of the primary surgery; Group B, revisions performed between two and five years after the primary surgery; and Group C, revisions performed more than five years following the primary procedure. In the Group A, N=50 (49%), the number of fusion and non-fusion index procedures was almost the same. However, in Group B (N=25, 24%), non-fusion procedures were more common than fusion procedures (n=14, 56%). In Group C, N=28 (27%), again the most common procedure done was a non-fusion procedure 20 (71%). The distribution of cases is shown in Figure 1.

The most common cause in group A patients was adjacent segment disease (ASD), in group B and group C it was recurrent stenosis. 

Among group A patients who required revision at the previously operated (index) level, 15 cases were identified as recurrent disc herniations and seven as residual disc herniations. These were determined based on a pain-free interval of less than six months following the index procedure. Seven patients with residual fragments had undergone micro lumbar discectomy (MLD) during the primary surgery, with one case initially treated using an endoscopic approach. In group B and C, all the cases were due to recurrent stenosis at the same level. In group A, patients who underwent revision after index fusion procedures, three implant-related cases were identified, two due to implant pull-out and one due to a symptomatic screw breach.

Statistical analysis of the dataset revealed no significant association between BMI and the occurrence of ASD following spinal fusion. The chi-squared test yielded a p-value of 0.815. The analysis shown in Table 2.

**Table 2:** Association of body mass index (BMI) with the development of adjacent segment disease (ASD) among patients undergoing revision lumbar spine surgery Data is presented as n (%). The cutoff for obesity was defined as BMI ≥30 kg/m². Statistical analysis was performed using the Chi-square test. A p-value of <0.05 was considered statistically significant. Despite a slightly higher incidence of ASD in the obese group (36.7%) compared to the non-obese group (34.2%), the difference was not statistically significant (p=0.815).

Category	BMI <30 (Non-obese)	BMI ≥30 (Obese)	Total
ASD present	25 (34.2%)	11 (36.7%)	36
ASD absent	48 (65.8%)	19 (63.3%)	67
Total	73	30	103

**Figure 2:**
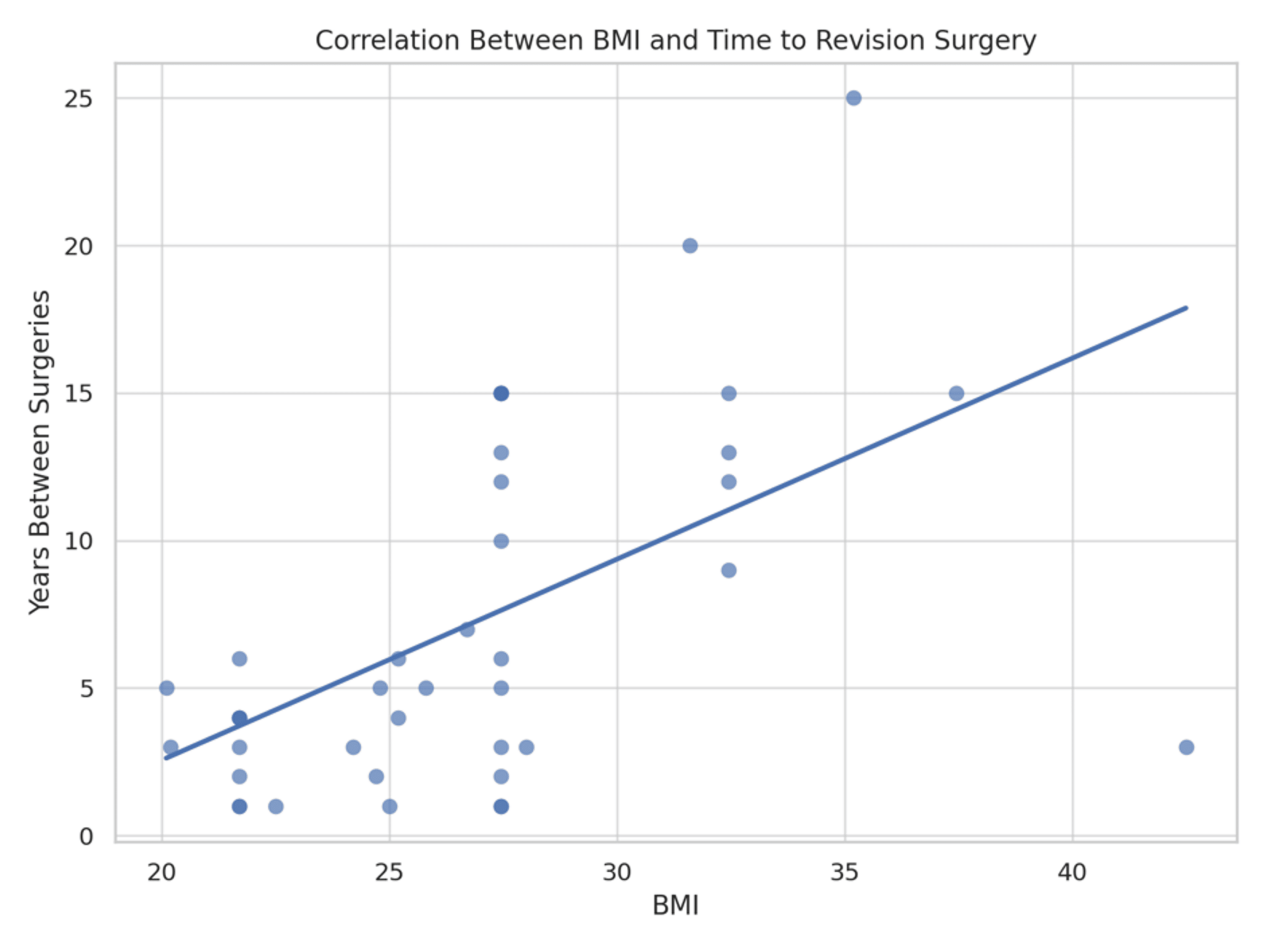
Scatter plot analysis correlating body mass index (BMI) with the time interval to revision surgery The scatter diagram depicts the distribution of 103 patients based on their BMI (kg/m²) along the horizontal axis and the duration from the index surgery to the revision procedure (in years) along the vertical axis. Spearman’s rank correlation analysis was utilized to assess the relationship between obesity and the longevity of the primary surgery. The analysis indicated moderate positive linear correlation between higher BMI and a shorter time interval to revision (p<0.12). Correlation coefficient r=0.15.

The analysis revealed a moderate positive correlation (r=0.15) between the BMI and the time interval between the primary and revision spinal surgeries. The scatter plot is shown in Figure 2.

No significant association was observed between age and the occurrence of ASD. Only 36 (34%) patients had their imaging from previous surgery available for analysis. Out of them, 18 cases were from Group A, 10 from Group B and eight from Group C.

Out of 103 revision surgeries, 21 had their index surgery at our institution. Among these, the etiology was ASD in nine cases (42.9%), implant loosening or failure with or without infection in five cases (23.8%), recurrent stenosis in two cases (9.5%), recurrent disc after endoscopy in two cases (9.5%), and adjacent vertebral fracture in two cases (9.5%). Implant loosening was due to an infection in four cases and osteoporosis in one case. Both the recurrent disc after endoscopy were reoperated within three months from primary surgery. The mean interval between surgeries was 5.6 years for ASD, 3.1 years for implant loosening, and 11.2 years for redo decompression.

**Figure 3:**
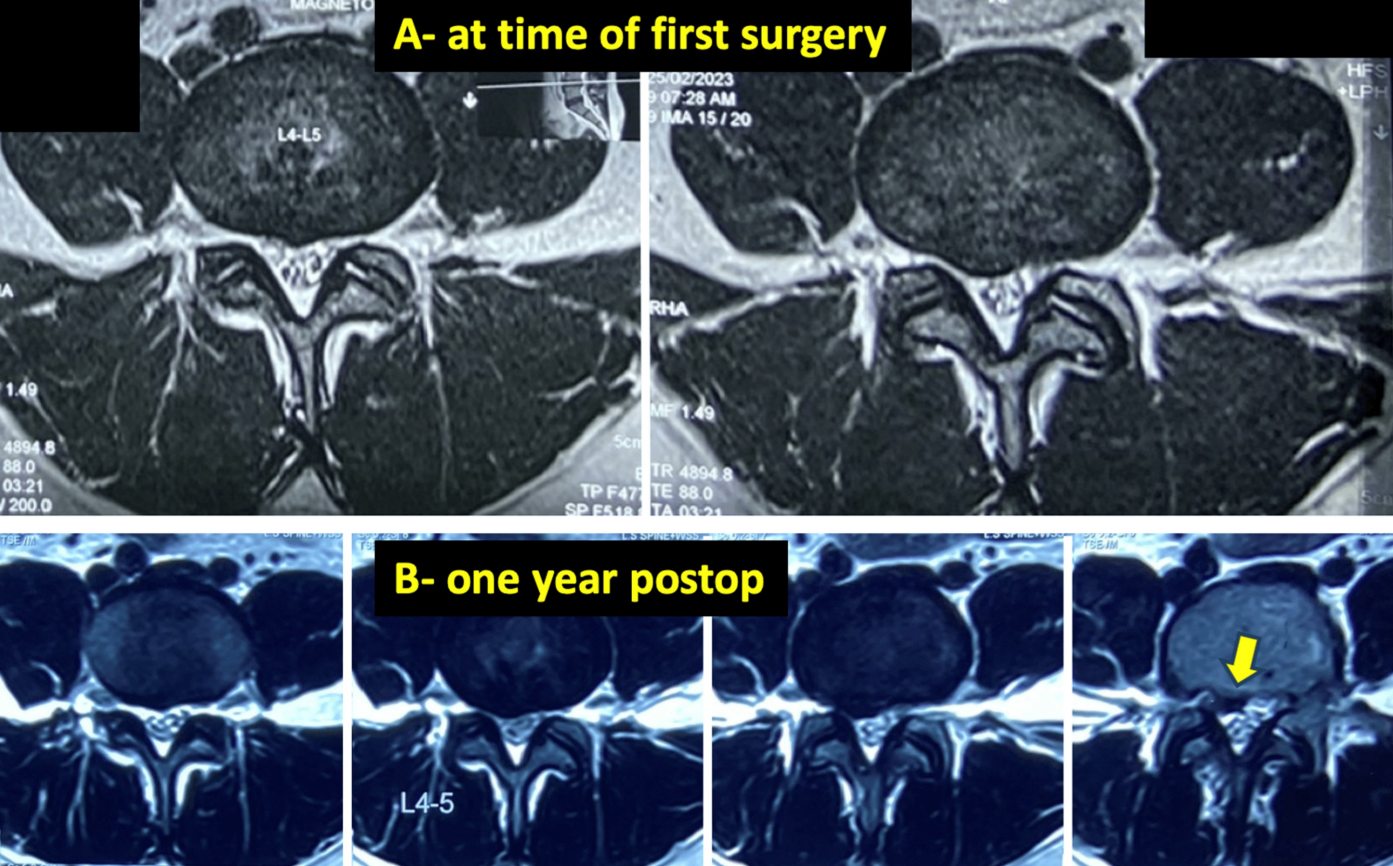
A 33-year-old female patient with recurrent disc requiring revision in a case of postoperative micro lumbar discectomy (MLD) A: preoperative imaging (axial T2 weighted axial MRI)  showing  paracentral disc bulge of L45 level addressed with micro lumbar decompression; B: one year postop imaging (axial T2 weighted axial MRI), showing recurrent disc at the same level which now is down migrated (yellow arrow).

**Figure 4:**
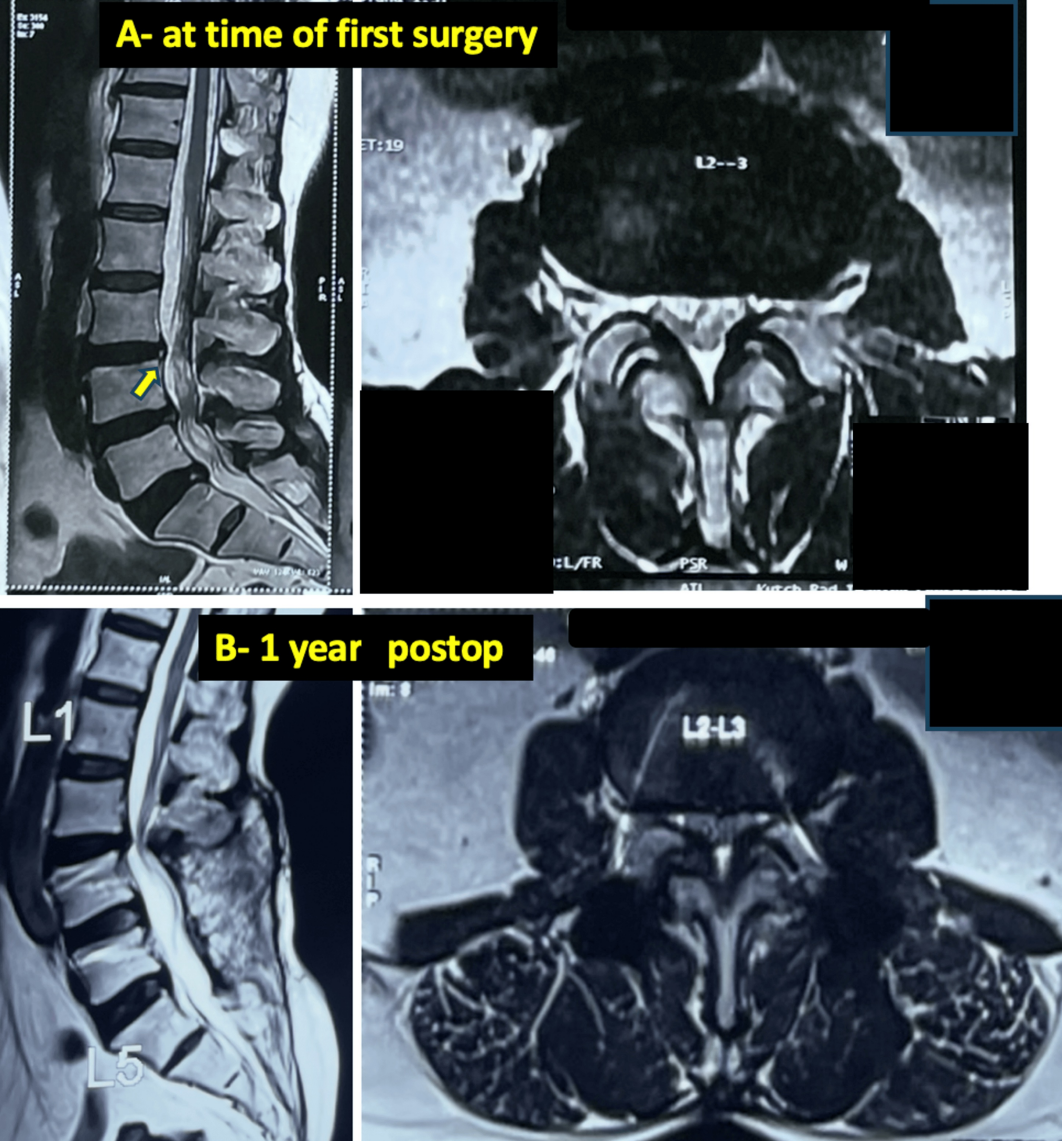
A 45-year-old male patient with ignored adjacent segment moderate stenosis which rapidly progressed after fusion leading to revision A: preoperative images of T2 weighted mid sagittal and axial images at the adjacent level in a case of L3-L4 stenosis operated with L34 fusion in the first surgery. Note the hyperintense zone (yellow arrow) at the L2-L3 level; B: One-year postoperative image of the same patient showing significant progression of the adjacent segment (L23) stenosis compared to the preop imaging within a short timespan.

**Figure 5:**
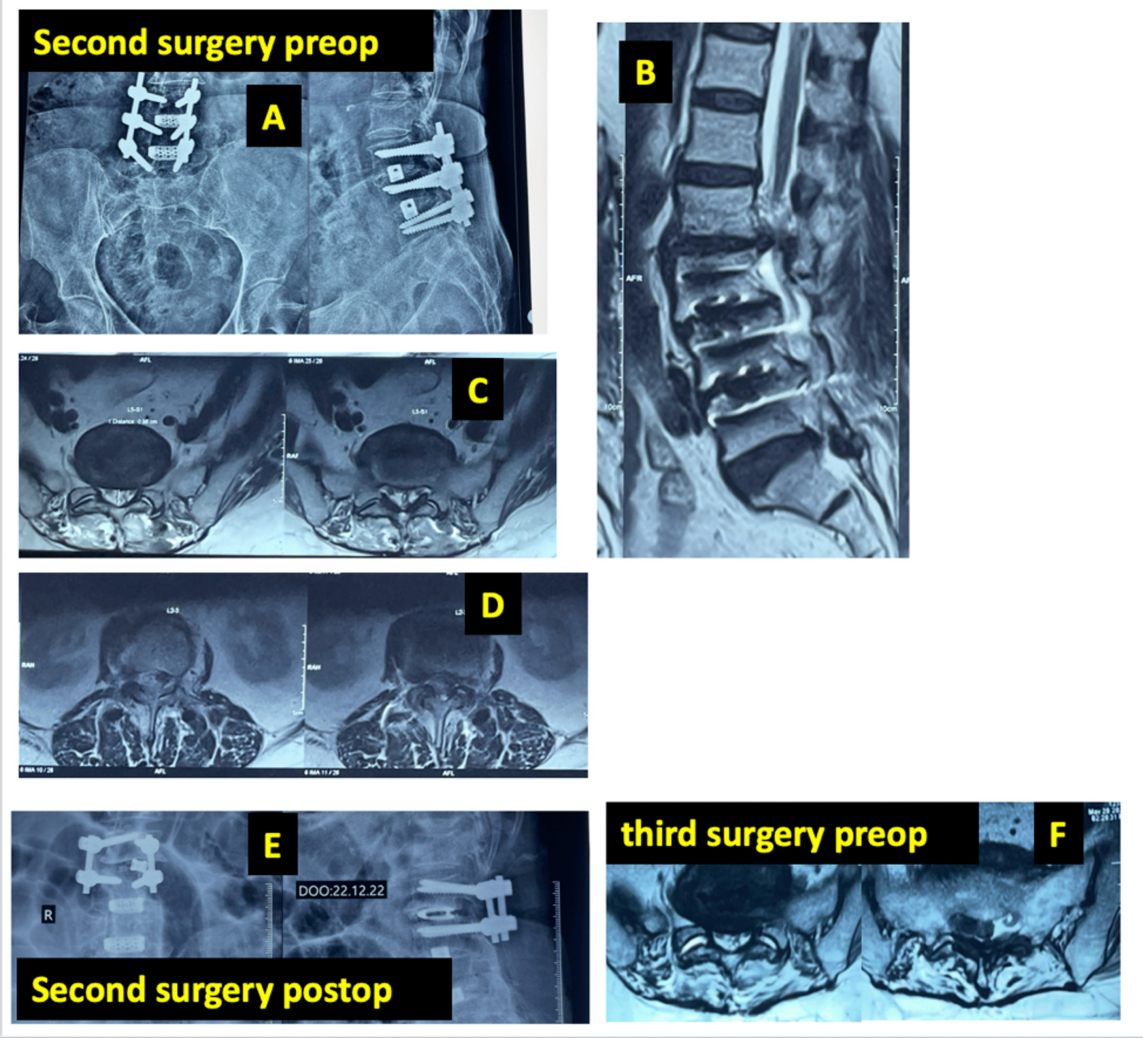
A 40-year-old male patient who underwent long fusion from L2-L5, developing a L5S1 prolapsed intervertebral disc (PID) after a two year follow-up A: Preoperative X-ray of the patient who developed adjacent segment disease (ASD) after L3-5 fusion; B, D: preoperative T2 weighted sagittal and axial MRI showing ASD at the above level during the first revision; C: axial T2 weighted MRI at the L5S1 showing healthy L5S1 at the time of first revision while addressing L23 ASD; E: postoperative X-ray after L23 fusion for the ASD; F: T2 weighted axial MRI imaging at two years after first revision showing a disc prolapse at the L5S1 level probably due to the increased stresses due to long fusion.

Examples of cases from Group A are shown in Figures 3-5.

**Figure 6:**
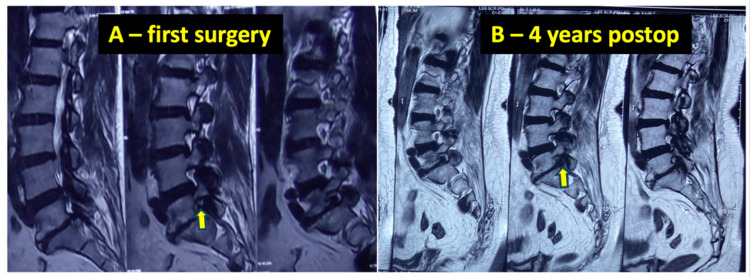
A 56-year-old male patient with inadequately addressed foraminal stenosis leading to revision A: Preoperative T2 weighted sagittal MRI showing central stenosis and asymptomatic foraminal stenosis (yellow arrow) addressed with central decompression only; B: Postoperative T2 weighted sagittal MRI taken four years later, when patient developed radiculopathy attributable to the previously asymptomatic foraminal stenosis (yellow arrow) which became symptomatic.

A example case from group B is shown in Figure 6.

An analysis of complications during revision surgery showed that five cases had dural tears (5%) during the revision procedure, and 13 cases had wound complications, out of which one had to undergo re-suturing after debridement.

A paired T-test was used for comparison between preoperative and six-month postoperative functional scores, and the results are summarized in Table 3.

**Table 3:** Statistical analysis of the improvement in the outcomes preoperatively to six months after the procedure (n=103) A paired t-test was used to compare preoperative and six-month postoperative scores for visual analog scale (VAS) back pain (VAS-BP), VAS leg pain (VAS-LP), and Oswestry Disability Index (ODI). A p-value <0.05 was considered statistically significant. Values of VAS and ODI given as mean (standard deviation).

Parameter	Preoperative (Mean ± SD)	Postoperative (Mean ± SD)	t-value	p-value
VAS-BP	8.32 ± 1.74	3.57 ± 1.23	26.4	<0.001
VAS-LP	7.31 ± 2.23	2.51 ± 2.10	22.8	<0.001
ODI	75.78 ± 11.38	25.82 ± 9.35	38.2	<0.001

There was a statistically significant improvement in pain and functional outcomes at the six-month follow-up. Mean VAS-back pain or VAS-BP decreased from 8.32 to 3.57, VAS-leg pain or VAS-LP from 7.31 to 2.51, and ODI from 75.78 to 25.82 (p<0.001 for all comparisons), indicating substantial postoperative improvement in both pain and disability.

## Discussion

Revision lumbar spine surgery is becoming an increasingly significant aspect of spinal care. At our tertiary referral center, a relatively stable volume of primary lumbar surgeries (1,209 in 2023 and 1,288 in 2024) was accompanied by a rise in revision surgeries (from 136 to 169), reflecting a 14.7% year-on-year increase. This disproportionate increase mirrors global trends as observed by Rajaee et al. (2012), who attributed the increased numbers to failures from primary surgeries, along with increased referral for complex revisions [13]. This surge, although partly attributable to improved surgical access and increased patient awareness, also raises concerns about inadequately planned or inappropriate initial interventions. Preventable causes such as infections and residual disc fragments can be significantly reduced through meticulous surgical technique and perioperative care.

Radiating leg pain and numbness were the most common presenting complaints at revision, consistent with findings from Khan et al. (2024) [14]. Studies on repeat decompression have shown that among common presenting complaints in failed back surgery, such as radiating leg pain and low back pain, patients with radiating pain supported by corresponding radiological findings responded favorably to revision decompression. Hence, appropriate case selection is crucial to achieving good surgical outcomes [15].

Group A

Early revision surgeries (within two years of the index procedure) were predominantly driven by technical factors, particularly in non-fusion cases. Residual disc fragments accounted for seven of the 24 non-fusion revisions in group A (14% of group A), underscoring the importance of meticulous preoperative MRI assessment and accurate intraoperative correlation of fragment size. Failure to correlate radiologic and intraoperative findings can result in incomplete fragment excision and persistent radiculopathy requiring reoperation.

Recurrent disc herniation was even more common in this group, occurring in 15 non-fusion patients (30%). Figure 3 illustrates one such case. Radiologic predictors like disc bulges (vs. extrusions) were commonly associated with revisions. Huang (2016) and Brooks (2021) noted increased recurrence for bulges after discectomy due to their early degenerative nature, and linked recurrence to Modic changes, disc height, and sagittal motion. This finding highlights the necessity for judicious case selection, particularly with regard to disc morphology, degree of degeneration, and patient-specific biomechanical factors [16,17].

In our group of early revisions, one case was due to recurrent disc herniation following transforaminal endoscopic discectomy (TFED). Recurrence rates in TFED have been studied and found to be 11% with high BMI and modic changes and disc protrusion type being factors associated with it [18,19].

Of note, seven out of the eight infection cases occurred in fusion patients, mostly with diabetes (six of eight; 75%). Previous studies identified instrumentation, prolonged operative time, need for blood transfusion, obesity, and diabetes as key risk factors for infection [20,21].

The risk of implant-related revision can be significantly reduced by the use of newer technologies like 3D navigation and intraoperative neuromonitoring (IONM) [22]. We encountered one case of screw breach (operated elsewhere) that required revision. Additionally, there were two cases of implant pull-out-one in an L1-S1 fusion without interbody fusion at the last level in a 65-year-old female patient, and another in an L4-S1 Transforaminal Lumbar Interbody Fusion or TLIF in a 75-year-old female patient. Both patients were osteoporotic, with Dual-Energy X-ray Absorptiometry or DEXA T-scores < -2.5 on preoperative assessment for revision surgery and they underwent extensions of fixation to the pelvis. Multiple studies aimed at preventing implant pull-out have emphasized that, in osteoporotic patients undergoing L5-S1 fusion, incorporating L5-S1 TLIF or using S2-alar-iliac or S2AI screws can improve construct stability. Adhering to principles such as screw medialization and the use of larger-diameter screws is also recommended [23,24].

In the early failures, five patients had Schizas grade B or C stenosis at adjacent levels during their primary surgery, which were inadequately decompressed. These segments rapidly progressed and required revision within five years. One such case is shown in Figure 4. Studies on the causes of such early ASD have found multi-level fusions to be one contributing factor; however, the five failures in our group included both fusion (three cases) and non-fusion (two cases) procedures in the primary surgery [25]. Liang et al. (2014) highlighted pre-existing adjacent disc degeneration as a strong predictor for such progression of ASD. Our study confirm these findings [26].

In our group of early revisions, an interesting case of neglected pseudomeningocele post-MLD at another institution was noted. Initially managed conservatively at our institute, the patient eventually had to undergo revision with fusion due to non-resolution of symptoms and the presence of an intraoperatively detected pars fracture (in the revision surgery). Management for such cases of postoperative pseudo meningocele includes surgical re-exploration or, in select cases, conservative modalities such as epidural blood patches, depending on the symptoms and defect size [27,28].

L5-S1 degeneration is common after long fusions ending at L5, particularly when preexisting L5-S1 disc degeneration or facet arthropathy is present. Several studies recommend extending fusion to the sacrum in such cases. However in our study among eight patients who underwent multi-level fusion in primary surgery (with L5S1 not included in the construct), only one (Figure 5) developed L5-S1 degeneration. Others showed proximal-level degeneration. All the seven cases had partially sacralized L5 vertebrae, offering stability at L5-S1 but shifting stress proximally, hence the conflicting results [29,30].

Group B

In Group B, the majority of cases were recurrence of stenosis in non-fusion patients N=12 (50%), underscoring the challenge of achieving lasting decompression without stabilization. Two ASD cases among the non-fusion patients suggested that while ASD is more prevalent post-fusion due to altered biomechanics, it also has a component of the natural history of disc aging at those adjacent segments [31].

From the primary surgery imaging that could be collected, one patient (Figure 6) was identified who had asymptomatic foraminal stenosis missed at index surgery, leading to symptoms four years later. Ikegami et al. (2016) found that 15% of such patients with initially neglected foraminal stenosis require revision within two years, especially when retrolisthesis is present. Our study supports addressing even asymptomatic foraminal stenosis if risk factors exist [32].

Group C

In group C (>5 years), ASD was the sole cause in fusion patients, while recurrent stenosis dominated non-fusion cases. Lee et al. (2014) reported a 10% ASD incidence over 10 years post-fusion [33]. Studies by Scemama et al. and Maruenda et al. estimate adjacent segment degeneration at 5% per year and ASD at 1.8% annually [34,35].

Analysis of the cases who had their primary surgery done at our institute showed that ASD was most common etiology with average time interval 5.6 years between the index fusion and revision and the cases who had index non fusion were less in number and had their redo decompression at 11.9 years on average. This discrepancy may be explained by the fact that our hospital is a tertiary referral center for spinal surgery. Many inadequately decompressed cases initially operated elsewhere undergo revision here, resulting in more redo decompressions for non-fusion cases in the overall cohort but fewer such cases among those originally operated at our institution.

While the present findings indicate no statistically significant relationship between BMI and ASD, previous studies have reported divergent conclusions. Some research suggests that elevated BMI increases mechanical stress on adjacent segments, accelerating degeneration and the likelihood of revision surgery. Conversely, other analyses demonstrate no consistent correlation between obesity and ASD risk, implying that multiple factors, such as sagittal imbalance, fusion length, and bone quality, play more dominant roles [26,36].

Complications were infrequent in our revision cohort. We recorded five dural tears (5%), all of them in multilevel revisions. Studies by Eichholz et al. (2013) and Melcher et al. (2022) indicate higher dural tear risks in revisions due to fibrosis and altered anatomy [5,37]. Baker et al. (2012) cited revision status, surgical invasiveness, age, and diabetes as predictors [38].

We only had one case of deep surgical site infection which was a case of multi-level fusion revision, suggesting increased risk with surgical complexity. Gerometta et al. (2012) and Peng et al. (2018) also found prolonged surgery, blood loss, obesity, and diabetes as key infection contributors [20,21].

At the six-month follow-up, significant VAS and ODI improvement in our patients demonstrated that revision surgery can yield favorable outcomes when indicated and well-planned. Although Evidence-Based Medicine (EBM) guidelines exist for conditions such as disc herniation and spinal stenosis, they are not consistently followed, often resulting in failed primary surgeries [7]. Non-adherence to these EBM protocols have been shown to necessitate repeated surgical interventions, which are associated with progressively reduced patient satisfaction [39]. Despite the North American Spine Society (NASS) advocacy for EBM in lumbar fusions, compliance remains low (60% as per Thomson et al., 2013) [7,40,41]. Psychological factors has been shown to influence recovery. Higher Zurich Depression Scores correlate with worse outcomes, indicating the need for preoperative mental health assessments and counseling. 

Fusion rates were not assessed in our study, but literature (Dede et al., 2013; Getzbein et al., 1998) reports 90-100% radiologic success [42,43]. However, radiologic fusion doesn’t always translate to clinical satisfaction; pseudarthrosis patients had only ~62% satisfaction despite fusion [42].

This study has certain limitations. It is a single-center study with a relatively short follow-up period of six months. There is also a selection bias because of tertiary referral setting. The etiological profile may vary across different centers, limiting the generalizability of the findings. Moreover, a six-month follow-up is insufficient to assess long-term outcomes or radiological fusion status. A two-year follow-up analysis of these patients is currently ongoing.

## Conclusions

The etiology of revision lumbar spinal surgery varies depending on the interval since the initial operation, with recurrent disc compression from incomplete decompression during primary surgery emerging as the most prevalent indication across all time groups. Notably, patients who had non-fusion procedures outnumbered those receiving fusion surgeries. The present study also revealed that overlooked diagnoses at the primary surgery, particularly ASD and foraminal stenosis, were frequent underlying causes for subsequent revision. These findings highlight the need for thorough preoperative assessment and meticulous surgical technique to minimize the risk of recurrence and additional intervention.
